# Effect of Rapid Maxillary Expansion on the Pterygoid Process and Spheno‐Occipital Synchondrosis in Skulls With Class II and Class III Skeletal Relationships: A Finite Element Analysis Study

**DOI:** 10.1155/ijod/5168602

**Published:** 2026-01-24

**Authors:** Manuel Gustavo Chávez-Sevillano, Alexandre Rodrigues Freire, Ana Cláudia Rossi, Cátia Cardoso Abdo Quintão, Felippe Bevilacqua Prado

**Affiliations:** ^1^ Department of Pediatric Stomatology, School of Dentistry, National University of San Marcos, Av. Germán Amézaga 375, Cercado de Lima-Lima, Lima, Peru, unmsm.edu.pe; ^2^ Department of Morphology, Piracicaba Dental School, State University of Campinas—UNICAMP, Anatomy Area, Avenue Limeira, 901, Piracicaba, São Paulo, Brazil, unicamp.br; ^3^ Department of Orthodontics, School of Dentistry, Rio de Janeiro State University, Boulevard 28 de Setembro, 157; Vila Isabel, Rio de Janeiro, Rio de Janeiro, Brazil, uerj.br

**Keywords:** finite element analysis, malocclusion, rapid maxillary expansion

## Abstract

**Objetive:**

To evaluate the effect of the rapid maxillary expansion (RME) on the pterygoid process (PP), spheno‐occipital synchondrosis (SOS), and sella turcica (ST) in two patients’ skulls with Class II and Class III skeletal relationships and to identify differences in mechanical loading through finite element analysis (FEA).

**Materials and Methods:**

Cone‐beam computed tomography (CBCT) scans of two patients’ skulls were used: (1) a 13‐year‐old female with a Class II skeletal relationship due to maxillary protrusion and (2) a 14‐year‐old male with a Class III skeletal relationship due to maxillary hypoplasia. The computer‐aided design (CAD) geometry of both skulls was imported into Ansys v14 software to construct the finite element mesh. A transverse force of 100 N was applied to the palatal surfaces of the first upper molar and first premolar to simulate RME. Von Mises stress (VMS) and maximum principal stress (MPS) were assessed at selected nodes representing the anatomical areas of interest.

**Results:**

In VMS, the Class II model showed the highest value at Point 3 (2.077 MPa), similar to the Class III model (1.707 MPa). In MPS, the highest tensile stress in the Class II model occurred at Point 2 (1.396 MPa), while in the Class III model it was at Point 3 (1.813 MPa).

**Conclusions:**

The PP, SOS, and ST in Class II and Class III skeletal patterns are subjected to tensile and compressive loads during RME. The Class III model exhibited higher stress distribution at the skull base compared with the Class II model.

**Clinical Significance:**

Understanding how delicate cranial structures respond to heavy orthopedic loads generated during RME may help refine treatment protocols for transverse maxillary deficiency across different malocclusions.

## 1. Introduction

Rapid maxillary expansion (RME) is widely used in children, adolescents, and even adults. It is commonly indicated for the treatment of posterior crossbite, maxillary atresia in skeletal Class II cases, and skeletal Class III correction combined with maxillary protraction [1–4]. The primary anatomical and biomechanical objective of RME is to open the midpalatal suture (MPTS), which becomes increasingly resistant with age. With the advent of cone‐beam computed tomography (CBCT), studies have demonstrated that ossification of the MPTS does not correlate strictly with chronological age [5–7].

Many young adult patients are now treated using bone anchorage through micro‐implant‐assisted rapid palatal expansion (MARPE) [8–10]. The stresses tolerated by craniofacial structures during RME have been previously documented [11–13]. Among the circummaxillary sutures offering the greatest resistance to MPTS opening are the zygomaticomaxillary and pterygopalatine sutures [14].

The maxilla is closely connected to the skull base through the pterygoid processes (PPs) of the sphenoid bone. Mechanical stress generated by expansion appliances during MPTS opening may therefore affect these structures [15]. Several studies have examined the mechanical effects of RME on the spheno‐occipital synchondrosis (SOS) [14, 16–20], the PP [16, 21–23], and the sella turcica (ST) [22, 24]. Some researchers have argued that the SOS plays a significant role in shaping craniofacial morphology during growth [25], suggesting that RME‐induced modifications may influence craniofacial development [26]. Conversely, other authors report that RME affects only the maxillary region of the skull base when orthopedic forces are applied [27].

Because conventional orthodontic examinations cannot adequately reveal the biomechanical effect of RME on craniofacial structures, finite element analysis (FEA) has been widely used for this purpose [11, 13, 20]. It has been suggested that as patients age, RME or MARPE may exert more pronounced and potentially harmful effects on the skull base, particularly on the PP and SOS, potentially altering skeletal equilibrium. Understanding these effects is important for monitoring treatment outcomes across different skeletal classifications.

The present study evaluates the influence of RME on the PP, SOS, and ST in two patients’ skulls—one with a Class II and one with a Class III skeletal relationship—using FEA to identify differences in mechanical loading patterns.

## 2. Materials and Method

This study was approved by the Ethics Committee of the Piracicaba Dental School, State University of Campinas (Protocol Number: 056/2013). The CBCT images were obtained from the Radiology Service database of the School of Dentistry, National University of San Marcos (UNMSM).

### 2.1. Computed Tomography and Modeling for Computer‐Aided Design (CAD) Geometry Acquisition

This computational and descriptive study compared two skulls converted into CAD models, to which forces simulating RME were applied. CBCT scans of two patients’ skulls were used: (1) a 13‐year‐old patient with a Class II skeletal relationship (ANB: 7^°^) due to maxillary protrusion and (2) a 14‐year‐old patient with a Class III skeletal relationship (ANB: −2^°^) due to maxillary hypoplasia (Figure 1). Both skulls presented complete permanent dentition and posterior dental crossbite. The CBCT images were acquired with a slice thickness of 0.25 mm.

Figure 1(A) Pretreatment lateral cephalometric radiograph of the Class II skeletal relationship. (B) Pretreatment lateral cephalometric radiograph of the Class III skeletal relationship.

**Figure:**
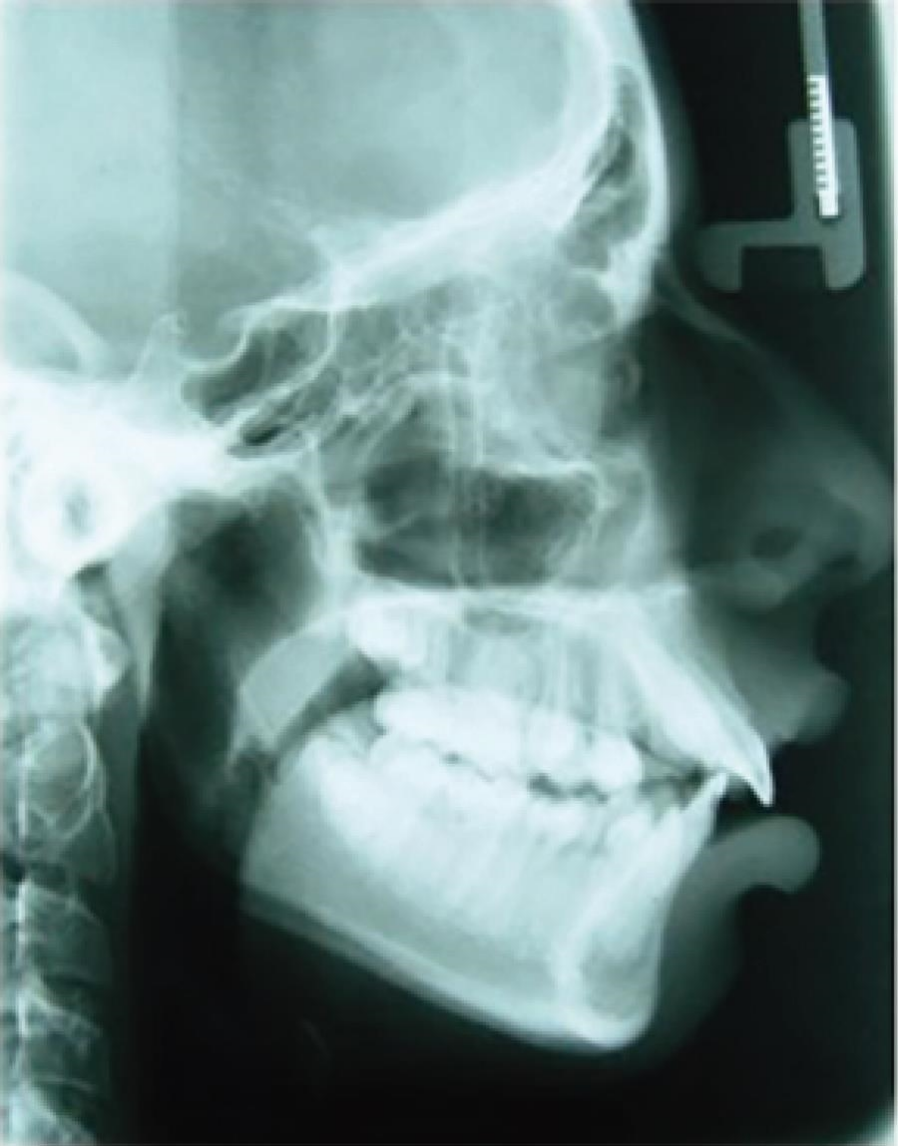
(A)

**Figure:**
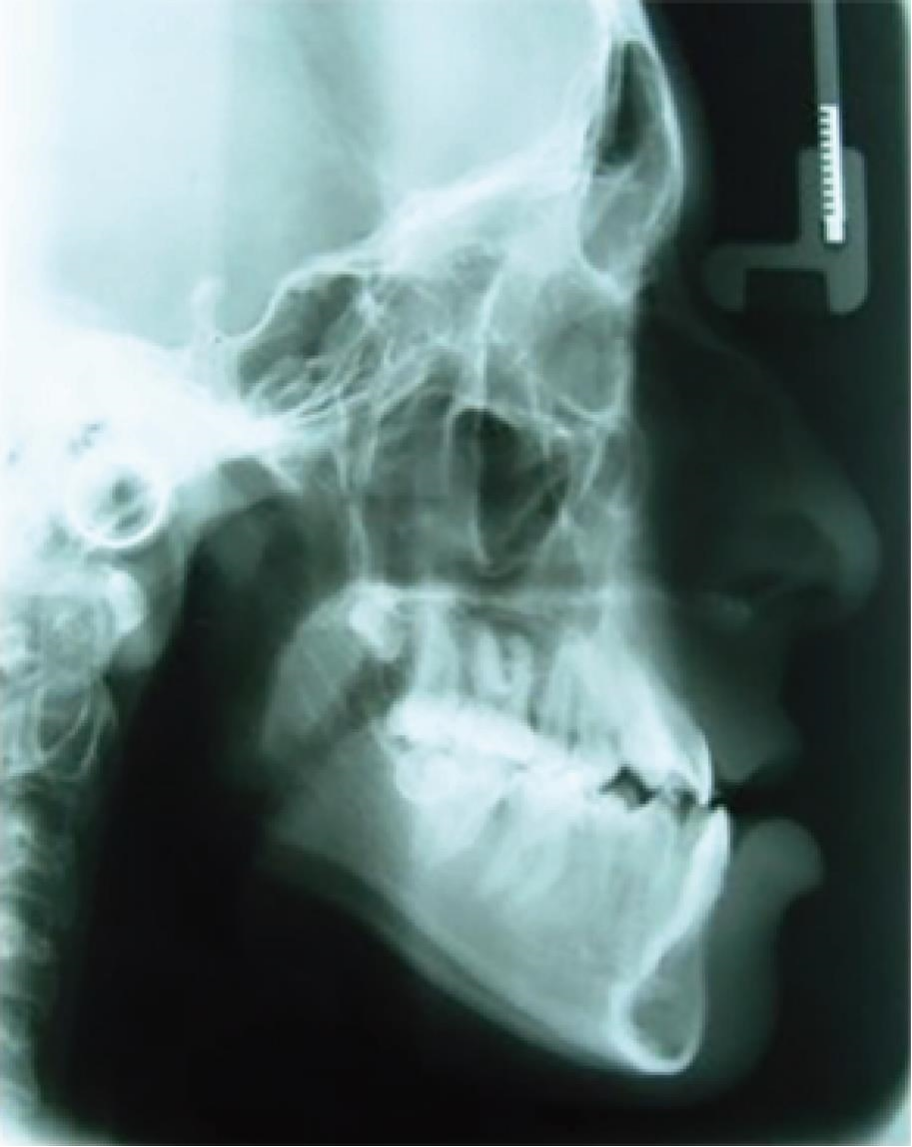
(B)

The CBCT images were imported into MIMICS v17 (Materialise, Belgium) and segmented to obtain the three‐dimensional surfaces of the maxilla and skull base. The selected anatomical structures were converted into three‐dimensional stereolithography (STL) surfaces. The CAD geometry was developed in Rhinoceros 5.0 (McNeel & Associates, Seattle, Washington; Figure 2). The modeling was performed by converting the STL surfaces into non‐uniform rational B‐spline surfaces. The spaces corresponding to the MPTS (red) and SOS (green) were filled with solid structures representing connective and cartilaginous tissues, respectively (Figure 3).

Figure 2(A) CBCT image showing selected anatomical structure. (B) CAD geometry created in Rhinoceros 5.0. (C) Finite element mesh generated in Ansys v14.

**Figure:**
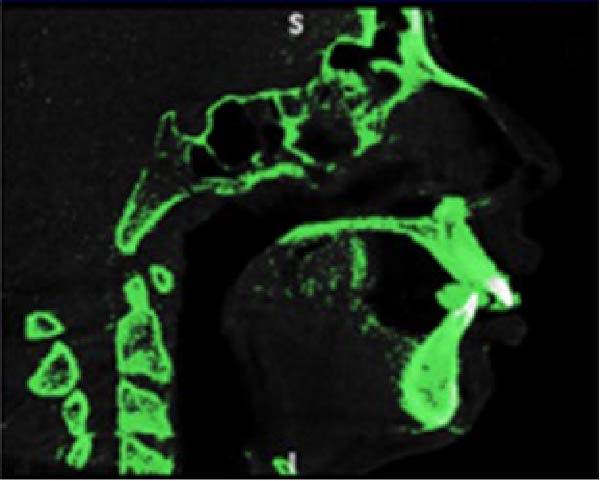
(A)

**Figure:**
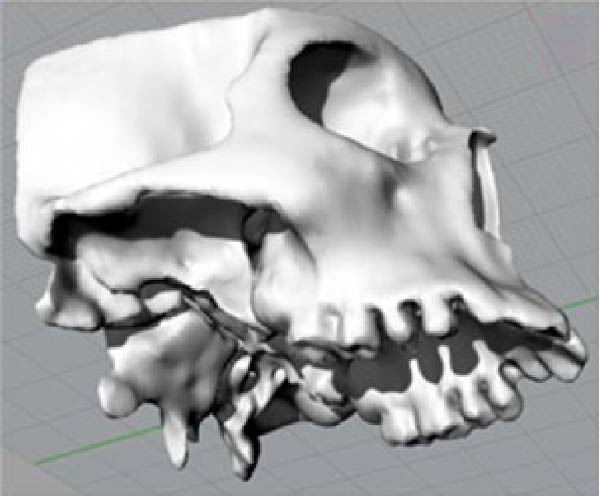
(B)

**Figure:**
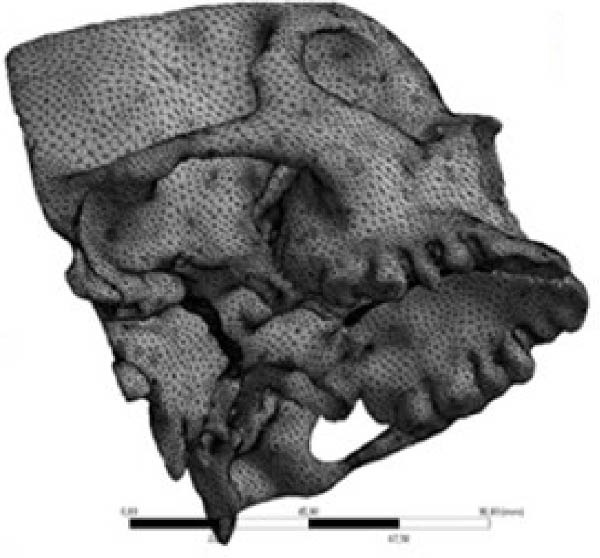
(C)

Figure 3Simulation of transverse loading (red arrows). (A) Lateral view of model. (B) Inferior view of model.

**Figure:**
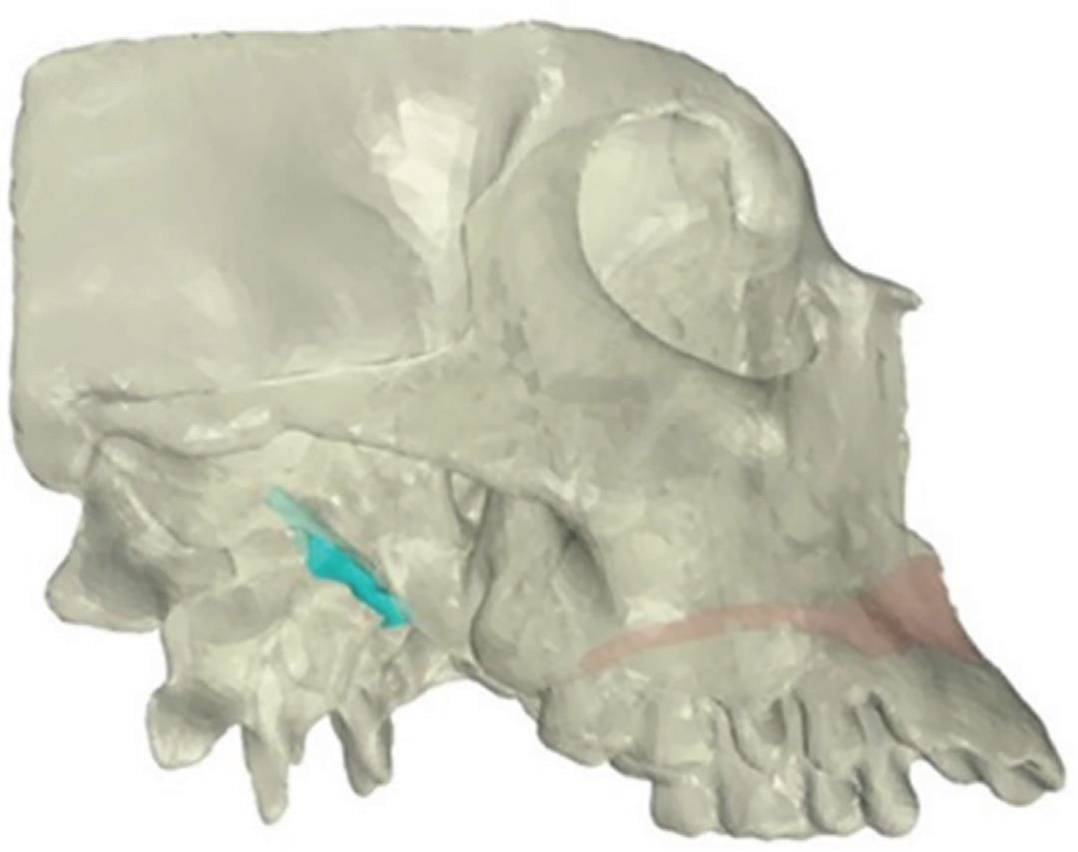
(A)

**Figure:**
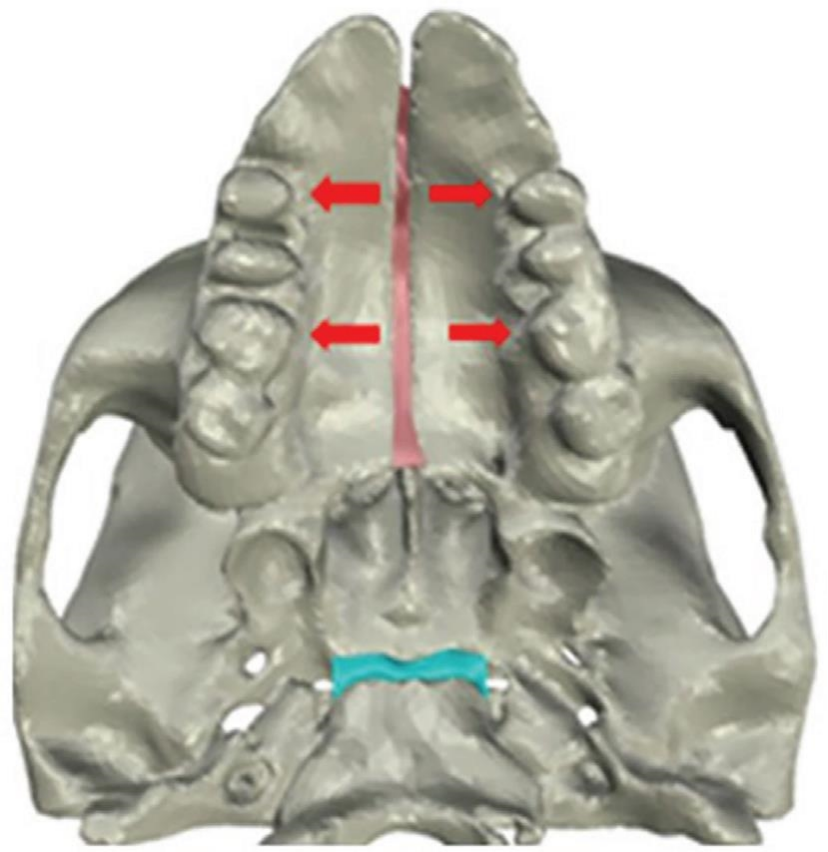
(B)

### 2.2. FEA

#### 2.2.1. Finite Element Model

The CAD geometries of two skulls were imported into Ansys v14 (Ansys, Inc., USA) to generate the finite element mesh (Figure 2). Tetrahedral elements were used, resulting in meshes containing 344,808 elements and 596,966 nodes for the Class II model and 390,349 elements and 689,736 nodes for the Class III model. Material properties were defined as linear elastic and isotropic. Bone, cartilage (for the SOS), and connective tissue (for the MPTS) were assigned material properties according to the previous studies [28–30] (Table 1).

**Table 1:** Mechanical properties used in the study.

Material	Young’s modulus (MPa)	Poisson’s ratio
Bone [28]	14,000	0.3
Midpalatal suture [29]	1	0.3
Spheno‐occipital synchondrosis [30]	24	0.3

#### 2.2.2. Boundary Conditions and Configuration of Analyses

Zero displacement and zero rotation were assigned to the nodes along the margin of the foramen magnum. The geometric configuration and applied loads were set symmetrically along the transverse (*X*) axis. To simulate RME, the force was applied not to the maxilla or alveolar process but directly to the teeth. A transverse force of 100 N [30] was applied to the palatal surfaces of the first upper molar and first premolar, following established protocols [31] (Figure 3). To visualize the biomechanical effects on the PP, SOS, and ST in Class II and Class III skulls, each finite element model was sectioned sagittally [15, 32].

#### 2.2.3. Analyses of Results

**Figure 4:**
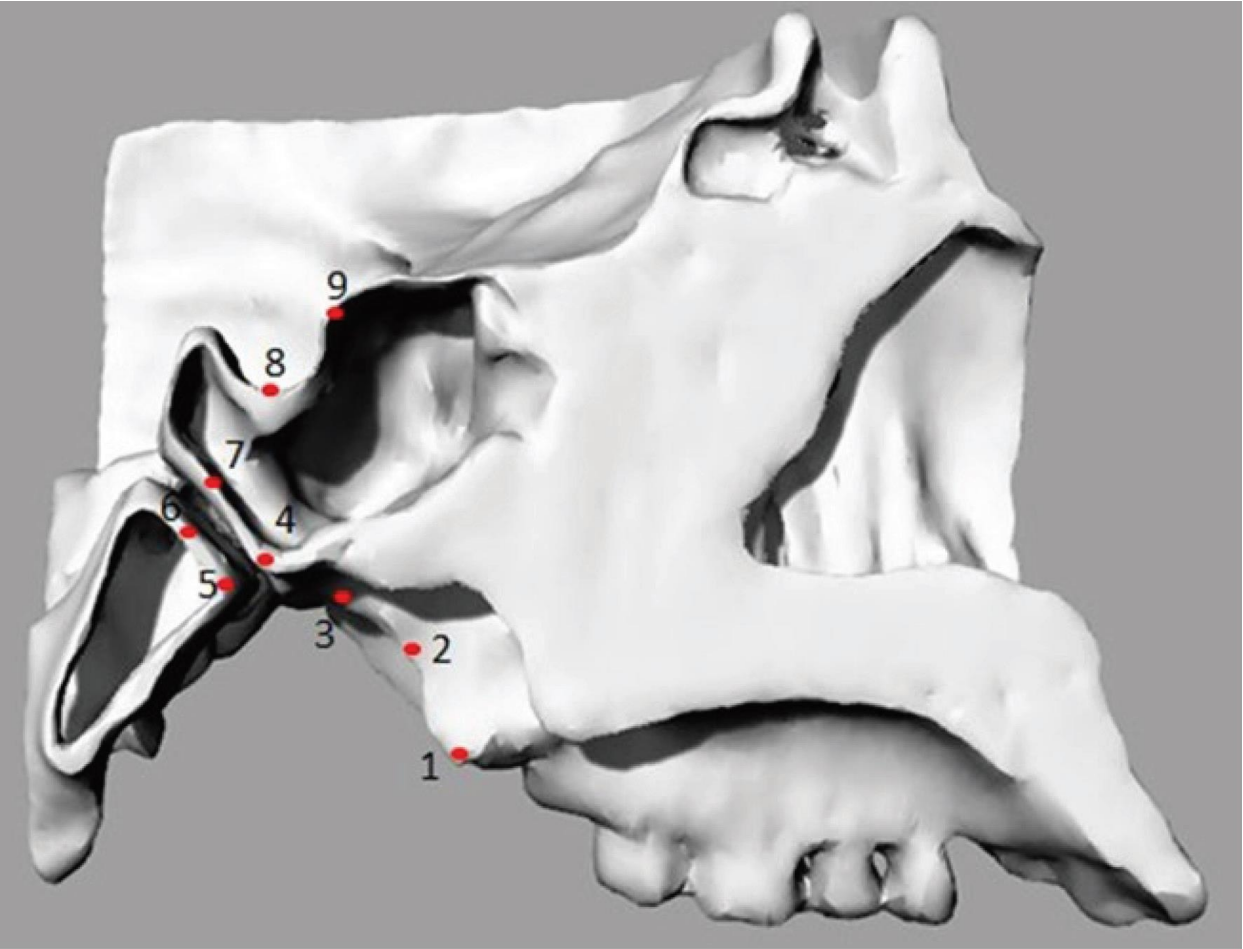
Points selected for stress evaluations in the sagittal view.

Von Mises stress (VMS) and maximum principal stress (MPS) were evaluated by identifying specific nodes, which were represented as points corresponding to key anatomical regions as described in a previous study [12]: sphenobasion anterior (Sba, Point 4), sphenobasion posterior (Sbp, Point 5), inferior SOS (SOi, Point 6), superior SOS (Sos, Point 7), sella (S, Point 8), and sphenoidal point (Sphen, Point 9). Additionally, three points were included to assess the medial pterygoid plate at the lower (Point 1), middle (Point 2), and upper (Point 3) levels (Figure 4 and Table 2).

**Table 2:** Points corresponding to the anatomical structures.

Points	Selected nodes on
Point 1	The most inferior and posterior point of the medial pterygoid plate
Point 2	The most posterior and middle point of the medial pterygoid plate
Point 3	The most upper and posterior point of the medial pterygoid plate
Point 4	The most inferior point of the posterior surface of the sphenoid body
Point 5	The most inferior point of the anterior surface of the basilar part of the occipital bone
Point 6	The point of intersection between the Sba line and the anterior surface of the basilar of the occipital bone
Point 7	The point of intersection between the Sba line and the posterior surface of the sphenoid body
Point 8	The deepest point of the floor of the sella turcica
Point 9	The uppermost point of the tuberculum sellae

FEA was performed on each sagittal section. Under the applied loading condition, VMS represented the effective stress on the material, while MPS indicated the tensile and compressive stresses in the anatomical region of interest.

## 3. Results

### 3.1. VMS Comparison Between Class II and Class III Skull Models

In the Class II model, the highest VMS value was observed at the Point 3 (2.077 MPa), similar to the Class III model (1.707 MPa). When comparing the two skeletal patterns, VMS increased along the medial pterygoid plate from the inferior to the superior region (Points 1, 2, and 3), with higher stress values observed in the Class II model than in the Class III model (Figure 5 and Table 3).

Figure 5VMS distribution showing overall stress concentration. (A) Class II model. (B) Class III model.

**Figure:**
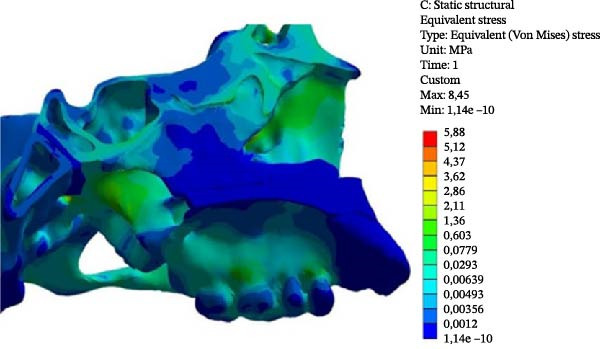
(A)

**Figure:**
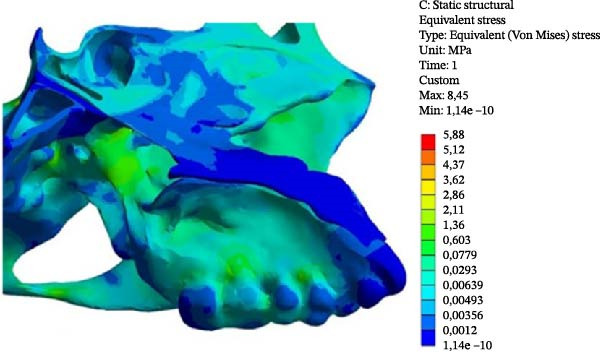
(B)

**Table 3:** VMS and MPS values at each anatomical points evaluated.

Point	Von Mises stress (MPa)		Maximum principal stress (MPa)	
	Class II	Class III	Class II	Class III
1	0.050	0.124	0.030	0.127
2	1.466	0.904	1.396	0.885
3	2.077	1.707	1.104	1.813
4	0.324	0.373	0.229	0.435
5	0.143	0.066	0.157	0.069
6	0.036	0.054	0.023	0.066
7	0.212	0.346	0.238	0.387
8	0.193	0.341	−0.008	−0.066
9	0.177	0.281	0.183	0.014

In both models, the anterior region of the SOS (Points 4 and 7) showed greater stress concentration than the posterior region (Points 5 and 6). VMS values in the anterior region were similar between the two models (Table 3).

At the ST (Point 8), the Class III model demonstrated greater stress (0.341 MPa) compared with the Class II model (0.193 MPa). At the tuberculum sellae (Point 9), the Class III model also showed higher stress (0.281 MPa) than the Class II model (0.177 MPa) (Figure 5).

### 3.2. MPS Comparison Between Class II and Class III Skulls Models

MPS values included both positive (tensile) and negative (compressive) stresses. The maximum tensile stress in the Class II model occurred at Point 2 (1.396 MPa), whereas, in the Class III model, it occurred at Point 3 (1.813 MPa; Table 3).

In both models, the anterior region of the SOS (Points 4 and 7) exhibited higher tensile stress compared with the posterior region (Points 5 and 6). Tensile stress values were consistently higher in the Class III model.

Compressive stress was identified at the ST (Point 8) and was greater in the Class III model (0.066 MPa) than in the Class II model (0.008 MPa). At the tuberculum sellae, tensile stress was higher in the Class II model (0.183 MPa) than in the Class III model (0.014 MPa; Figure 6).

Figure 6MPS distribution showing tensile (red, orange, and yellow) and compressive (blue and light blue) stresses. (A) Class II model. (B) Class III model.

**Figure:**
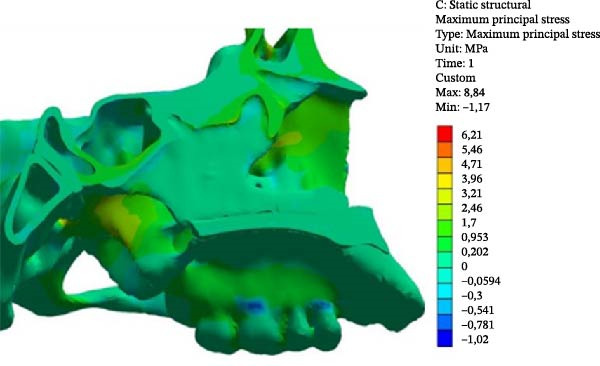
(A)

**Figure:**
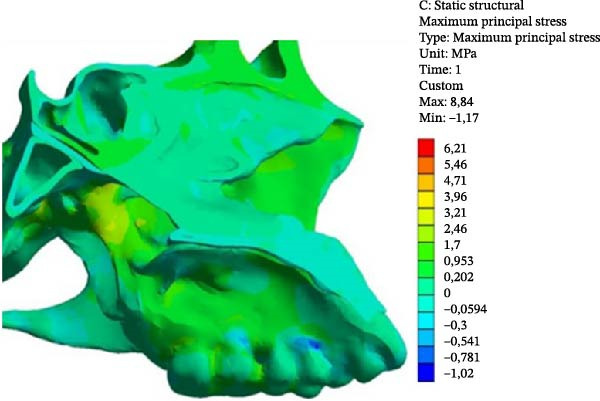
(B)

## 4. Discussion

The CBCT scans used in this study were obtained from an existing database, and the stages of MPTS maturation in both skulls were assessed using the protocol of Angelieri et al. [5]. Both skulls were classified as stage “C,” indicating that the MPTS was not fully ossified. Skeletal maturity was evaluated following Baccetti et al. [33], revealing that the Class II patient was at stage CSIII and the Class III patient at stage CSIV, confirming that both were in active growth. Because this was a computational study, the differences in stress distribution between Class II and Class III skulls were primarily influenced by morphological variations, as geometry strongly affects mechanical behavior [28].

RME is widely used to correct transverse discrepancies in both Class II and Class III skeletal relationships. Conventional RME and MARPE promote MPTS opening, and transverse forces may be transmitted to the skull base through the maxilla–PP connection [18, 25]. Some authors suggest that failure to open the pterygopalatine suture during expansion may increase stress transmission to the skull base, especially in older patients [15].

Various biomechanical effects during RME have been documented, including displacement of the SOS [18, 21, 22], changes in bone metabolism [16, 34], and alterations in the anterior cranial base. Skull base morphology plays a crucial role in determining sagittal jaw relationship [35], yet the influence of RME on skull base mechanics in Class II and III cases remains insufficiently explored [36].

Because palatal displacement was not simulated, only the first stage of RME—lateral force application—was examined, following the methodology of Boryor et al. [30, 31] and Lee et al. [37]. Consistent with previous studies, our results showed higher stress concentration in the anterior SOS (Points 4 and 7). Gardner and Kronman [38] suggested that stress in this region may contribute to maxillary displacement during the active phase of RME.

In MPS analysis, tensile stress appeared in both models. In the Class II model, it was greatest in the middle region of the medial pterygoid plate (Point 2), whereas, in the Class III model, it appeared at the superior region (Point 3). These findings align with Holberg et al. [39], who observed similar stress distribution patterns, likely due to lateral bending of the PP during RME, especially when the pterygopalatine suture does not open [11, 15, 21].

Recent studies by Liu et al. [4] and Shi et al. [23] demonstrated displacements and stresses at the PP and skull base in Class III patients undergoing RME, supporting our findings. Cho et al. [10] and Leonardi et al. [20] also reported SOS changes during expansion, indicating its biomechanical sensitivity to RME.

Compressive stress was also observed at the ST (Point 8), with higher values in the Class III model. Holberg et al. [39] noted compressive stress at the SOS during RME, and Thilander and Ingervall [40] described a collagen fiber pattern at the SOS that may facilitate tensile stress distribution. The differences in tensile and compressive patterns around the SOS in this study may reflect variations in tissue elasticity [31].

The tuberculum sellae (Point 9) showed greater tensile stress in the Class II model. According to Afrand et al. [34], the anterior cranial base is not a stable reference structure, as the ST undergoes remodeling during growth. This remodeling may influence stress absorption during RME. Additionally, cartilage persistence in the ST of older individuals may contribute to reduced stress levels [40].

This study assumed isotropic and linearly elastic skull structures [41], which represents a limitation. Differences in stress distribution were largely morphology‐dependent, and the values reported are not absolute [15]. Moreover, structures such as teeth, periodontal ligament, cancellous bone, and circummaxillary sutures were not individually segmented. Points selected for stress assessment were based on prior research examining SOS response to RME [15].

Clinically, it has been suggested that fractures occurring during expansion of interdigitated sutures—and their subsequent healing—may restrict growth and predispose patients to bone defects [26]. Based on our computational findings, older patients and those with Class III skeletal patterns may experience greater stress on the PP, SOS, and skull base during RME. The Class III model showed particularly high stress values, likely related to reduced cranial base length or bone quantity, suggesting a greater predisposition to microfractures and structural compromise.

Further studies, including mechanical testing and clinical evaluation, are needed to refine RME protocols considering age, maturation, and desired expansion. This study contributes to understanding the biomechanical impact of expansion forces on delicate craniofacial structures.

## 5. Conclusion

• The PP, SOS, and ST in Class II and Class III skeletal patterns are subjected to tensile and compressive loads during RME.
• The Class III model exhibited higher distribution at the skull base compared with the Class II model.
• Stress on the SOS and ST was consistently greater in the Class III model.

## Author Contributions

**Manuel Gustavo Chávez-Sevillano:** conceptualization, methodology, writing – original and final draft preparation, responsible for manuscript submission and responding to reviewers’ comments. **Alexandre Rodrigues Freire and Ana Cláudia Rossi:** methodology, software, project administration and interpretation of the data. **Cátia Cardoso Abdo Quintão:** writing – reviewing and editing. **Felippe Bevilacqua Prado:** writing – original draft preparation and supervision.

## Funding

This article was supported in part by the Coordinação de Aperfeiçoamento de Pessoal de Nível Superior‐Brazil (CAPES)—(Finance Code 001).

## Disclosure

This research is part of the thesis submitted for a Master’s Degree in Oral Biology, which can be found at the following links: https://repositorio.unicamp.br/Acervo/Detalhe/957754 file:///C:/Users/Preethi.N/Downloads/chavezsevillano_manuelgustavo_m.pdf

## Ethics Statement

This study was approved by the Ethics Committee of Piracicaba Dental School, State University of Campinas (Protocol Number: 056/2013).

## Conflicts of Interest

The authors declare no conflicts of interest.

## Data Availability

The data that support the findings of this study are available from the corresponding author upon reasonable request.

## References

[1] Hass A. J. , Rapid Expansion of the Maxillary Dental Arch and Nasal Cavity by Opening the Mid-Palatal Suture, The Angle Orthodontist. (1961) 31, no. 2, 73–90.

[2] Krebs A. , Midpalatal Suture Expansion Studies by the Implant Method Over a Seven-Year Period, Transaction European Orthodonthic Society. (1964) 40, 131–142. 14318002

[3] Garret B. J. , Caruso J. M. , Rungcharassaeng K. , Farrage J. R. , Kim J. S. , and Taylor G. D. , Skeletal Effects to the Maxila After Rapid Maxillary Expansion Assessed With Cone-Beam Computed Tomography, American Journal of Orthodontics and Dentofacial Orthopedics. (2008) 134, no. 1, 8–9. 18617096 10.1016/j.ajodo.2008.06.004

[4] Liu Y. , Feng F. , Wang Y. , Chi J. , and Liu W. , Three-Dimensional Displacement of Partial Craniofacial Bones Following Rapid Maxullary Expansion in Young Patients With Angle Class III Malocclusions, Journal of Craniofacial Surgery. (2019) 30, no. 4, 1004–1008, 10.1097/SCS.0000000000005417, 2-s2.0-85067465981. 30817506

[5] Angelieri F. , Cevidanes L. H. S. , Franchi L. , Gonçalves J. R. , Benavides E. , and McNamara Jr J. A. , Midpalatal Suture Maturation: Classification Method for Individual Assessment Before Rapid Maxillary Expansion, American Journal of Orthodontics and Dentofacial Orthopedics. (2013) 144, no. 5, 759–769, 10.1016/j.ajodo.2013.04.022, 2-s2.0-84887114817. 24182592 PMC4185298

[6] Chávez-Sevillano M. G. , Estrada J. T. , Blanco-Victorio D. J. , Vich M. O. L. , Quintão C. C. A. , and Palomino-Gómez S. P. , Evaluation of the Suture Ossification Level According to Age and Sex, in Children, Adolescents, and Adults. A Cross-Sectional and Observational 3D Study, International Orthodontics. (2021) 19, no. 1, 67–75, 10.1016/j.ortho.2020.12.002. 33531276

[7] Gokturk M. and Yavan M. A. , Comparison of the Short-Term Effects of Tooth-Bone-Borne and Tooth-Borne Rapid Maxillary Expansion in Older Adolescents, Journal of Orofacial Orthopedics/Fortschritte der Kieferorthopädie. (2024) 85, no. 1, 43–55, 10.1007/s00056-022-00401-x. 35612594

[8] Carlson C. , Sung J. , McComb R. W. , Machado A. W. , and Moon W. , Microimplant-Assisted Rapid Palatal Expansion Appliance to Orthopedically Correct Transverse Maxillary Deficiency in an Adult, American Journal of Orthodontics and Dentofacial Orthopedics. (2016) 149, no. 5, 716–728, 10.1016/j.ajodo.2015.04.043, 2-s2.0-84969520066. 27131254

[9] Brunetto D. P. , Sant’Anna E. F. , Machado A. W. , and Moon W. , Non-Surgical Treatment of Transverse Deficiency in Adults Using Microimplant-Assisted Rapid Palatal Expansion (MARPE), Dental Press Journal of Orthodontics. (2017) 22, no. 1, 110–125, 10.1590/2177-6709.22.1.110-125.sar, 2-s2.0-85018691670. 28444019 PMC5398849

[10] Cho M. H. , Choi Y.-K. , Kim S.-H. , Kim S.-S. , Park H. R. , and Kim Y.-I. , Effect of Miniscrew-Assisted Maxillary Protraction on Width Changes in the Circummaxillary Sutures, The Angle Orthodontist. (2025) 95, no. 3, 283–289, 10.2319/081424-665.1. 39970941 PMC12017546

[11] Işeri H. , Tekkaya A. E. , Öztan Ö. , and Bilgiç S. , Biomechanical Effects of Rapid Maxillary Expansion on the Craniofacial Skeleton, Studied by the Finite Element Method, European Journal of Orthodontics. (1998) 20, no. 4, 347–356, 10.1093/ejo/20.4.347, 2-s2.0-0032134301. 9753816

[12] Jafari A. , Shetty K. S. , and Kumar M. , Study of Stress Distribution and Displacement of Various Craniofacial Structures Following Application of Transverse Orthopedic Forces—A Three-Dimensional FEM Study, Angle Orthodontist. (2003) 73, no. 1, 12–20. 12607850 10.1043/0003-3219(2003)073<0012:SOSDAD>2.0.CO;2

[13] Moon W. , Wu K. W. , and MacGinnis M. , et al.The Efficacy of Maxillary Protraction Protocols With the Micro-Implant-Assisted Rapid Palatal Expander (MARPE) and the Novel N2 Mini-Implant—A Finite Element Study, Progress in Orthodontics. (2015) 16, no. 1, 1–14, 10.1186/s40510-015-0083-z, 2-s2.0-84982867223. 26061987 PMC4456601

[14] Ghoneima A. , Abdel-Fattah E. , Hartsfield J. , El-Bedwehi A. , Kamel A. , and Kula K. , Effects of Rapid Maxillary Expansion on the Cranial and Circummaxillary Sutures, American Journal of Orthodontics and Dentofacial Orthopedics. (2011) 140, no. 4, 510–519, 10.1016/j.ajodo.2010.10.024, 2-s2.0-80053518314. 21967938 PMC5161454

[15] Sevillano M. G. C. , Kemmok D. T. , Noritomi P. Y. , Fernandes L. Q. P. , Junior J. C. , and Quintão C. , New Highlights on Effects of Rapid Palatal Expansion on the Skull Base: A Finite Element Analysis Study, Dental Press Journal of Orthodontics. (2021) 26, no. 6, 10.1590/2177-6709.26.6.e2120162.oar, e2120162. 34932710 PMC8690457

[16] Baydas B. , Yavuz I. , Uslu H. , Dagsuyu I. M. , and Ceylan I. , Nonsurgical Rapid Maxillary Expansion Effects on Craniofacial Structures in Young Adult Females, The Angle Orthodontist. (2006) 76, no. 5, 759–767. 17029507 10.1043/0003-3219(2006)076[0759:NRMEEO]2.0.CO;2

[17] Leonardi R. , Cutrera A. , and Barbato E. , Rapid Maxillary Expansion Affects the Spheno-Occipital Synchondrosis in Youngsters, The Angle Orthodontist. (2010) 80, no. 1, 106–110, 10.2319/012709-56.1, 2-s2.0-70449382384. 19852648 PMC8978747

[18] Silvestrini-Biavati A. , Angiero F. , Gambino A. , and Ugolini A. , Do Changes in Spheno-Occipital Synchondrosis After Rapid Maxillary Expansion Affect the Manxillomandibular Complex?, European Journal of Paediatric Dentistry. (2013) 14, no. 1, 63–67. 23597224

[19] Dong Z. , Shang J. , Sun Y. , and Gou N. , Evaluation of the Effect of Two Types of Arch Expansion Comnibed With Maxillary Protraction in the Treatment of Class III Bony Malformations, Shanghai Kou Qiang Yi Xue = Shanghai Journal of Stomatology. (2025) 34, no. 4, 440–443. 41157984

[20] Leonardi R. , Ronsivalle V. , Lagravere M. O. , Barbato E. , Isola G. , and Giudice A. L. , Three-Dimensional Assessment of the Spheno-Occipital Synchondrosis and Clivus After Tooth-Borne and Bone-Borne Rapid Maxillary Expansion: A Retrospective CBCT Study Using Voxel-Based Superimposition, The Angle Orthodontist. (2021) 91, no. 6, 822–829, 10.2319/013021-86.1. 34129666 PMC8549551

[21] Jafari A. , Shetty K. S. , and Kumar M. , Study of Stress Distribution and Displacement of Various Craniofacial Structures Following Application of Transverse Orthopedic Forces—A Three-Dimensional FEM Study, The Angle Orthodontist. (2003) 73, no. 1, 12–20. 12607850 10.1043/0003-3219(2003)073<0012:SOSDAD>2.0.CO;2

[22] Holberg C. and Rudzki-Janson I. , Stress at the Cranial Base Induced by Rapid Maxillary Expansion, The Angle Orthodontist. (2006) 76, no. 4, 543–550. 16808557 10.1043/0003-3219(2006)076[0543:SATCBI]2.0.CO;2

[23] Shi Y. , Zhu C.-N. , and Xie Z. , Displacement and Stress Distribution of the Maxilla Under Different Surgical Conditions in Three Typical Models With Bone-Borne Distraction: A Three-Dimensional Finite Element Analysis, Journal of Orofacial Orthopedics/Fortschritte der Kieferorthopädie. (2020) 81, no. 6, 385–395, 10.1007/s00056-020-00251-5. 33034698

[24] Sevillano M. G. C. , Effect of the Palatal Expansion on the Pterygoid Process, Spheno-Occipital Synchondrosis and Sella Turcica in Skulls With Class II and Class III Skeletal Relationship by Finite Element Analysis (FEA)”, 2015, State University of Campinas – UNICAMP.

[25] Kerr W. J. S. and Adam C. P. , Cranial Base and Jaw Relationships, American Journal of Physical Anthropology. (1988) 77, no. 2, 213–220, 10.1002/ajpa.1330770209, 2-s2.0-0023754506. 3207170

[26] Melsen B. and Ghafari J. G. , Myth and Evidence in Palatal Expansion, Seminars in Orthodontics. (2023) 29, no. 3, 278–288, 10.1053/j.sodo.2023.04.003.

[27] Jeffery N. , Cranial Base Angulation and Growth of the Human Fetal Pharynx, The Anatomical Record Part A: Discoveries in Molecular, Cellular, and Evolutionary Biology. (2005) 284A, no. 1, 491–499, 10.1002/ar.a.20183, 2-s2.0-18244377739. 15803472

[28] Wroe S. , Ferrara T. L. , McHenry C. R. , Curnoe D. , and U.Chamoli U. , The Craniomandibular Mechanics of Beging Human, Proccedings of the Royal B: Biological Sciences. (2010) 277, no. 1700, 3579–3586. 10.1098/rspb.2010.0509 PMC2982237 20554545

[29] Verrue V. , Dermaut L. , and Verhegghe B. , Three-Dimensional Finite Element Modeling of a Dog Skull for the Simulation of Initial Orthopeadic Displacements, The European Journal of Orthodontics. (2001) 23, no. 5, 517–527, 10.1093/ejo/23.5.517, 2-s2.0-0035487553. 11668871

[30] Boryor A. , Geiger M. , and Hohmann A. , et al.Stress Distribution and Displacement Analysis During An Intermaxillary Disjunction—A Three-Dimensional FEM Study of a Human Skull, Journal of Biomechanic. (2008) 41, no. 2, 376–382, 10.1016/j.jbiomech.2007.08.016, 2-s2.0-38149009588. 17949727

[31] Priyadarshini J. , Mahesh C. M. , Chandrashekar B. S. , Sundara A. , Arun A. V. , and Reddy V. , Stress and Displacement Patterns in the Craniofacial Skeleton With Rapid Maxillary Expansion—A Finite Element Method Study, Progress in Orthodontics. (2017) 18, no. 1, 10.1186/s40510-017-0172-2, 2-s2.0-85023763172, 17. 28603805 PMC5502214

[32] Trojan L. C. , Gonzáles-Torres L. A. , Melo A. C. , and de las Casas E. B. , Stress and Strain Analysis Using Different Palatal Expander Appliances in Upper Jaw and Midpalatal Suture, Artificial Organs. (2017) 41, no. 6, 1–11. 10.1111/aor.12817 27925236

[33] Baccetti T. , Franchi L. , and McNamara J. A.Jr., The Cervical Vertebral Maturation (CVM) Method for the Assessment of Optimal Treatment Timing in Dentofacial Orthopedics, Seminars in Orthodontics. (2005) 11, no. 3, 119–129, 10.1053/j.sodo.2005.04.005, 2-s2.0-24344460889.

[34] Afrand M. , Ling C. P. , Khosrotehrani S. , Flores-Mir C. , and Lagravère-Vich M. O. , Anterior Cranial-Base Time-Related Changes: A Systematic Review, American Journal of Orthodontics and Dentofacial Orthopedics. (2014) 146, no. 1, 21–32.e6, 10.1016/j.ajodo.2014.03.019, 2-s2.0-84903636845. 24974995

[35] Hopkin G. B. , Houston W. J. , and James G. A. , The Cranial Base as an Aetiological Factor in Malocclusion, The Angle Orthodontist. (1968) 38, no. 3, 250–255. 5242886 10.1043/0003-3219(1968)038<0250:TCBAAA>2.0.CO;2

[36] Maestripieri M. , Passaleva S. , Patanè B. , Cozzani P. , and Giorgetti R. , Functional-Orthopaedic Therapy and Cranial Base: Induced Changes, Utopia or Reality?, Progress in Orthodontics. (2002) 3, no. 1, 6–11, 10.1034/j.1600-9975.2002.00018.x.

[37] Lee H. , Ting K. , Nelson M. , Sun N. , and Sung S.-J. , Maxillary Expansion in Customized Finite Element Method Models, American Journal of Orthodontics and Dentofacial Orthopedics. (2009) 136, no. 3, 367–374, 10.1016/j.ajodo.2008.08.023, 2-s2.0-69249244280. 19732671

[38] Gardner G. E. and Kronman J. H. , Cranioskeletal Displacements Caused by Rapid Palatal Expansion in the Rhesus Monkey, American Journal of Orthodontics and Dentofacial Orthopedics. (1971) 59, no. 2, 146–155, 10.1016/0002-9416(71)90046-7, 2-s2.0-0015010203. 4993115

[39] Holberg C. , Steinhauser S. , and Rudzki-Janson I. , Rapid Maxillary Expansión in Adults: Cranial Stress Reduction Depending on the Extend of Surgery, The European Journal of Orthodontics. (2007) 29, no. 1, 31–36, 10.1093/ejo/cjl067, 2-s2.0-33846981529. 17290014

[40] Thilander B. and Ingervall B. , The Human Spheno-Occipital Synchondrosis II. A Histological and Microradiography Study of Its Growth, Acta Odontologica Scandinavica. (1973) 31, no. 5, 323–334, 10.3109/00016357309002520, 2-s2.0-0015730208. 4520245

[41] Sun W. , Starly B. , Nam J. , and Darling A. , Bio-CAD Modeling and Its Applications in Computer-Aided Tissue Engineering, Computer-Aided Design. (2005) 37, no. 11, 1097–1114, 10.1016/j.cad.2005.02.002, 2-s2.0-19044368357.

